# Sudden Sensorineural Hearing Loss May Increase the Risk of Retinal Vein Occlusion: A Nationwide Cohort Study

**DOI:** 10.3390/healthcare10020408

**Published:** 2022-02-21

**Authors:** Jong-Yeup Kim, Inseok Ko, Dong-Kyu Kim

**Affiliations:** 1 Department of Biomedical Informatics, College of Medicine, Konyang University, Daejeon 35365, Korea; jykim@kyuh.ac.kr (J.-Y.K.); sweetino@snu.ac.kr (I.K.); 2 Department of Otorhinolaryngology-Head and Neck Surgery, College of Medicine, Konyang University, Daejeon 35365, Korea; 3 Division of Big Data and Artificial Intelligence, Chuncheon Sacred Heart Hospital, Chuncheon 24253, Korea; 4 Department of Otorhinolaryngology-Head and Neck Surgery, Chuncheon Sacred Heart Hospital, Hallym University College of Medicine, Chuncheon 24253, Korea

**Keywords:** sudden sensorineural hearing loss, retinal vein occlusion, cohort study, incidence

## Abstract

Sudden sensorineural hearing loss (SSNHL) is thought to be a vascular disease. Retinal vein occlusion (RVO) is an also common ophthalmic vascular disease. Thus, we investigated the potential relationship between these using a retrospective nationwide cohort dataset. We compared 49,584 subjects in the SSNHL and the comparison (non-SSNHL) groups using patients randomly selected via propensity-score matching. We calculated the incidence, survival rate, and hazards ratio (HR) using log-rank test, and Cox proportional hazards regression models. This study examined a total of 375,490.4 person-years in the SSNHL group and 373,698.2 person-years in the comparison group. We found that 673 patients in the SSNHL group (1.8 cases per 1000 person-years) and 592 in the comparison group (1.6 cases per 1000 person-years) developed RVO during the 8-year follow-up period. The adjusted HR of RVO was 1.13 (95% confidence interval [CI] 1.01–1.26). The adjusted HR of developing RVO in SSNHL was the greatest in elderly patients (adjusted HR, 1.21; 95% CI, 1.01–1.46) and male patients (adjusted HR, 1.18; 95% CI, 1.03–1.34). Our findings suggest that clinicians should remain vigilant of the possibility of RVO development in SSNHL patients, specifically elderly male patients.

## 1. Introduction

Sudden sensorineural hearing loss (SSNHL) is characterized by abrupt unilateral loss of hearing, sometimes accompanied by tinnitus or vertigo [1]. It is characterized by acute dysfunction of the inner ear and affects men and women almost equally, with peak age-related incidence occurring between 50 and 60 years [2,3,4]. For most patients, their cases are regarded as idiopathic SSNHL because the etiology cannot be determined. However, some studies have suggested that idiopathic SSNHL has a vascular etiology [5,6,7]. The labyrinthine artery supplies the inner ear; it is a secondary branch of the anterior inferior cerebellar branch of the internal carotid artery. The relative lack of collateral blood supply in the inner ear contributes to ischemic vulnerability. Thus, studies defining risk factors for ischemic vascular disease, such as cigarette smoking and hypertension, also describe risk factors for the development of SSNHL [8,9,10,11]. Additionally, some cohort studies have shown that SSNHL is associated with a significant increase in risk of ischemic cardio-cerebrovascular diseases [12,13,14]. However, there have been few reports to date on the association between SSNHL and other vascular diseases.

Retinal vein occlusion (RVO) is a common ophthalmic vascular disease. RVO comprises a heterogeneous group of disorders with impaired venous return from retinal circulation [15]. It is usually classified into branch RVO and central RVO, depending on the site of obstruction [16]. Prevalence of RVO ranges from approximately 0.1%–0.6% with equal incidence between the sexes and increasing risk with older age [17,18,19]. The major risk factor for RVO is the occurrence of thrombophilia-related diseases, including hypertension, arteriosclerosis, and diabetes [15]. Several cohort studies have found that RVO is associated with a significant increase in the risk of stroke, heart failure, and acute myocardial infarction [20,21,22]. Despite this, the relationship between SSNHL and RVO has not been thoroughly investigated. Therefore, in the present study, we aimed to investigate the possible relationship between SSNHL and RVO and potential underlying factors, using a nationwide representative cohort dataset based on the Korea National Health Insurance Service (KNHIS-NSC).

## 2. Materials and Methods

### 2.1. Study Population and Independent Variables

This KNHIS database included all medical service utilization history for a representative sample of 1,025,340 South Koreans from 2002–2013. In this study, the SSNHL group included patients who were diagnosed with SSNHL (H912, H9120, H9121, H9129, or H810) between January 2002 and December 2005. In this study, to further improve the accuracy of the SSNHL definition, we exclusively included patients who had been diagnosed with SSNHL between 2002 and 2004 and were diagnosed by otorhinolaryngologists. Then, we excluded patients diagnosed with RVO (H34.1, H34.8) or patients who died of any cause between 2002 and 2005. Furthermore, we selected RVO events diagnosed by ophthalmologists. The comparison group was selected (one patient for each SSNHL patient) using propensity-score matching, according to age, sex, residential area, household income, disability, and comorbidities. We selected 49,584 eligible SSNHL patients and 49,584 non-SSNHL subjects. Each patient was followed up until 31 December 2013, or the occurrences of RVO. The study endpoints were defined as all-cause mortality or the diagnosis of RVO and if patients had no events until December 31, 2013, we censored.

### 2.2. Statistical Analysis

Incidence rates (per 1000 person-years) of RVO were investigated regarding the comparison groups. To identify differences in overall disease-free survival, Kaplan–Meier analysis was performed. In the subgroup analysis, we divided participants for comparison and SSNHL groups by age category or sex, and then these participants in each comparison and SSNHL groups were matched within the same age category or sex, and we compared the risk of RVO between comparison and SSNHL groups. Additionally, to detect whether SSNHL increased the risk of RVO occurrence, we calculated the hazard ratio (HR) and 95% confidence intervals (CI) using Cox proportional hazards regression analyses with a 2-sided significance level of *p* < 0.05. We used R version 4.3.1 (R Foundation for Statistical Computing, Vienna, Austria) on all statistical analyses.

## 3. Results

The present study included 49,584 patients with SSNHL and 49,584 without. We identified 375,490.4 person-years in the SSNHL group and 373,698.2 person-years in the comparison group, to evaluate RVO events. The characteristics of the study populations for the two cohorts (the SSNHL group and the comparison group) are presented in Table 1. We found that there were similar distributions of sex, age, residential area, household income, disability, and comorbidities between the two groups (*p* > 0.05). It means that all variables were well-matched.

**Figure 1:**
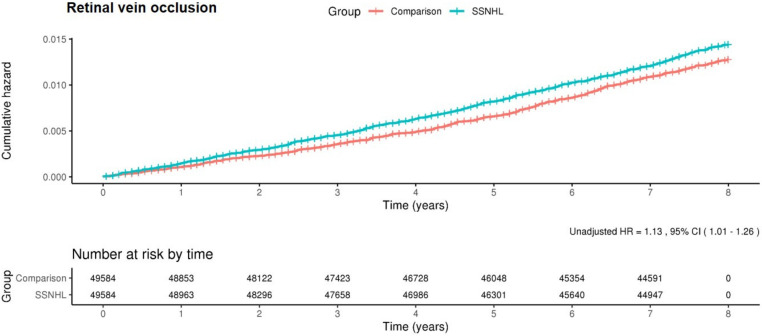
Kaplan–Meier survival curves and log-rank tests for RVO development in patients with SSNHL. (SSNHL—sudden sensorineural hearing loss; RVO—retinal vein occlusion).

To analyze the HRs for the incidence of RVO events during the 8-year follow-up period, we performed simple and multiple Cox regression models (Table 2). The SSNHL group showed significantly higher RVO incidence than the comparison group (1.8 per 1000 person-years in the SSNHL group and 1.6 per 1000 person-years in the comparison group) and the adjusted HR of RVO events in the SSNHL group was 1.13 (95% CI, 1.01–1.26). Figure 1 presents the overall disease-free events during the follow-up period using Kaplan–Meier survival curves with log-rank tests. The results of the log-rank test indicated that patients with SSNHL showed more frequent development of RVO events than controls.

When we performed the subgroup analysis according to age, a higher incidence of RVO was observed in the elderly group (3.3 per 1000 person-year in SSNHL and 2.7 per 1000 person-years in comparison). Additionally, the adjusted HR for developing RVO among the elderly with SSNHL during the 8-year follow-up period was 1.21 (95% CI: 1.01–1.46) (Table 3). In the other two age groups, there was no significant difference in the overall incidence of RVO between the SSNHL and non-SSNHL patients. Moreover, when we calculated the HRs for the development of RVO during the 8-year follow-up period by sex, we also observed that, after adjusting for other factors, male patients with SSNHL were more likely to develop RVO (adjusted HR 1.18 [95% CI 1.03–1.34]) compared to male subjects in the non-SSNHL group (Table 4). Conversely, there was no significant difference in RVO incidence between the SSNHL and non-SSNHL groups in female subjects.

## 4. Discussion

The present study used a nationwide retrospective cohort sample that many other studies have already used and published with [22,23,24,25]. Thus, the usage of this could eliminate selection bias and provide the chance to investigate the possible link between SSNHL and risk of RVO. To our knowledge, this is the first study to investigate a possible link between SSNHL and RVO. This study revealed an increased incidence of RVO in SSNHL patients compared to matched subjects. Interestingly, we also found that elderly or male patients with SSNHL showed an increased risk of RVO development than those with non-SSNHL. These findings suggest that physicians may conduct specific precautions to reduce the risk of RVO development among patients with SSNHL.

To date, various studies have proposed a relationship between SSNHL and vascular or hematologic pathologies [26,27,28]. These pathologies include emboli, transient ischemic attacks, sickle cell anemia, macroglobulinemia, and decreased blood supply to the cochlea, which reduces intracochlear oxygen levels, resulting in either transient or permanent hearing loss. One meta-analysis reported that cardiovascular risk factors appear to be associated with an increased risk of developing SSNHL [5]. Specifically, thrombophilic factor mutations also appear to increase the risk of SSNHL, especially in patients with a strong family history of thromboembolic events [26,27,28]. Several studies have also demonstrated that stroke risk is significantly associated with SSNHL [12,13,14]. Meanwhile, RVO pathogenesis is also associated with vascular problems. It generally develops because of compression of the venous lumen by arterial hemodynamic alterations, such as thrombosis [16]. Thus, RVO is more likely to occur in people with hypertension, high cholesterol levels, or other health problems that affect blood flow. Several studies described that coronary heart disease, such as myocardial infarction, because of its association with cardiovascular risk factors may be an independent risk factor of RVO [29,30,31]. Moreover, previous studies showed increased fibrinogen–albumin ratio defined as a marker of inflammation and disease severity in each patient with RVO or SSNHL [32,33]. One case study also reported the patients who suffered from central retinal vein occlusion and sudden deafness [34]. Collectively, these studies implied the possible link between SSNHL and RVO.

Generally, the peak incidence of SSNHL occurs between the fifth and sixth decades of life, although individuals of all ages can be affected [5,35]. Additionally, advanced age has been consistently identified as a major risk factor for RVO [17,29]. Old age can also be a source of traditional cardiovascular risk factors, such as hypertension or diabetes mellitus. Thus, to investigate the effect of age on SSNHL, and the subsequent development of RVO, we performed subgroup analysis according to age groups. We detected the risk of subsequent development of RVO during the 8-year follow-up period was greater in elderly patients with SSNHL (adjusted HR 1.21, 95% CI (1.01–1.46)) than in other age groups. Meanwhile, most studies have shown equal incidence in men and women [2,3,4], although one study described a slight female predominance in SSNHL, with a male-to-female ratio of 1:1.35 [36]. Additionally, several studies on RVO have described no predisposition for sex or ethnicity [15,37]. Interestingly, in this study, we found a significant likelihood of prospective development of RVO in male patients with SSNHL. Thus, we need further study on whether sex differences could influence the potential for microvascular damage.

Our study had several strengths. First, we used large national population-based data that were previously confirmed to provide for an effective analysis of specific disease incidence in South Korea [38]. Thus, the derived data are considered reliable. Secondly, to reduce the possibility of misdiagnosis, patients with SSNHL diagnosed by otorhinolaryngologists and patients with RVO diagnosed by ophthalmologists were included in this study. Despite its strengths, the study has some limitations. First, although we adjusted comorbidities as confounding variables, including hypertension, diabetes mellitus, and chronic renal failure, we could not obtain the specific health-related data regarding thrombophilic factors (e.g., body mass index, lipid profiles, smoking history, and alcohol consumption). Thus, we did not adjust these potential confounding factors in the present study. Additionally, systemic hypoxic damage conditions such as sleep apnea could up-regulate the local inflammation [39]. However, these variables could not be controlled in this study. Second, the diagnosis of disease based on the diagnostic code might be less accurate than the diagnosis obtained from a medical record that, for example, includes medical history, audiometry results, or fundoscopic images. Third, the KNHIS-NSC database provides categorized age data (<45, 45–64, and >64 years old). That’s why we could not match the two groups according to the actual age distribution and our findings may have some residual bias within the categories. Finally, due to the retrospective cohort design of the present study, we were unable to determine whether the relationship between SSNHL and RVO had the underlying mechanisms or was just a temporal incident. Also, to confirm the relationship between SSNHL and RVO, we need to investigate whether patients with RVO could increase the risk of developing SSNHL. Thus, further studies are warranted to confirm our findings conditions.

## 5. Conclusions

The present study investigated a possible relationship between SSNHL and the development of RVO during an 8-year follow-up period. We found that patients with SSNHL had a higher risk of developing RVO, with the risk being greater for elderly or male patients with SSNHL. However, further studies are needed to elucidate the underlying pathophysiological mechanisms. Therefore, this study provided new insight into the association between SSNHL and an ophthalmologic condition.

## Figures and Tables

**Table 1:** Characteristics of study subjects.

Variables	Comparison (*n* = 49,584)	SSNHL (*n* = 49,584)	*p* Value
**Sex**			0.984
Male	16,641 (33.6%)	16,645 (33.6%)	
Female	32,943 (66.4%)	32,939 (66.4%)	
**Ages (years)**			0.995
<45	19,375 (39.1%)	19,373 (39.1%)	
45–64	18,787 (37.9%)	18,777 (37.9%)	
>64	11,422 (23.0%)	11,434 (23.1%)	
**Residence**			0.999
Seoul (metropolitan)	9402 (19.0%)	9399 (19.0%)	
Second area (Other metropolitan)	12,598 (25.4%)	12,595 (25.4%)	
Third area	27,584 (55.6%)	27,590 (55.6%)	
**Household income**			0.999
Low (0–30%)	10,211 (20.6%)	10,214 (20.6%)	
Middle (30–70%)	18,011 (36.3%)	18,003 (36.3%)	
High (70–100%)	21,362 (43.1%)	21,367 (43.1%)	
**Disability**			1.000
No	47,612 (96.0%)	47,613 (96.0%)	
Yes	1972 (4.0%)	1971 (4.0%)	
**Comorbidities**			0.970
No	25,276 (51.0%)	25,269 (51.0%)	
Yes	24,308 (49.0%)	24,315 (49.0%)	

SSNHL—sudden sensorineural hearing loss; Seoul—the largest metropolitan area; second area—other metropolitan cities; third area—other areas.

**Table 2:** Incidence per 1000 person-years and hazard ratios for retinal vein occlusion.

Variables	N	Case	Incidence	Unadjusted HR (95% CI)	Adjusted HR (95% CI)
**Group**					
Comparison group	49,584	592	1.6	1 (ref)	1 (ref)
SSNHL group	49,584	673	1.8	1.13 (1.01–1.26) *	1.13 (1.01–1.26) *
**Sex**					
Male	33,286	390	1.6	1 (ref)	1 (ref)
Female	65,882	875	1.7	1.1 (0.98–1.24)	1.05 (0.93–1.18)
**Ages (years)**					
<45	38,748	112	0.4	1 (ref)	1 (ref)
45–64	37,564	693	2.4	6.61 (5.42–8.08) ***	4.88 (3.96–6.03) ***
>64	22,856	460	3	8.52 (6.93–10.48) ***	5.73 (4.59–7.16) ***
**Residence**					
Seoul	18,801	212	1.5	1 (ref)	1 (ref)
2nd area	25,193	297	1.6	1.06 (0.89–1.26)	1.07 (0.9–1.28)
3rd area	55,174	756	1.8	1.24 (1.06–1.44) **	1.16 (0.99–1.35)
**Household income**					
Low (0–30%)	20,425	274	1.8	1 (ref)	1 (ref)
Middle (30–70%)	36,014	458	1.7	0.93 (0.8–1.08)	1.04 (0.9–1.21)
High (70–100%)	42,729	533	1.7	0.92 (0.79–1.06)	0.94 (0.82–1.09)
**Disability**					
No	95,225	1196	1.7	1 (ref)	1 (ref)
Yes	3943	69	2.5	1.52 (1.19–1.93) **	1.14 (0.89–1.46)
**Comorbidities**					
No	50,545	306	0.8	1 (ref)	1 (ref)
Yes	48,623	959	2.7	3.53 (3.11–4.02) ***	1.91 (1.66–2.19) ***

SSNHL, sudden sensorineural hearing loss; HR, hazard ratio; CI, confidence interval. * *p* < 0.05, ** *p* < 0.010, and *** *p* < 0.001.

**Table 3:** Hazard ratios of RVO by age between patients with SSNHL and comparison (subjects without SSNHL).

Age (Years)	<45		45–64		>64	
	Comparison	SSNHL	Comparison	SSNHL	Comparison	SSNHL
uHR (95% CI)	1.00 (ref)	0.87 (0.6–1.25)	1.00 (ref)	1.12 (0.97–1.3)	1.00 (ref)	1.21 (1.01–1.46) *
aHR (95% CI)	1.00 (ref)	0.86 (0.6–1.25)	1.00 (ref)	1.12 (0.97–1.3)	1.00 (ref)	1.21 (1.01–1.46) *

SSNHL—sudden sensorineural hearing loss; RVO—retinal vein occlusion; uHR—unadjusted hazard ratio; aHR—adjusted hazard ratio; CI—confidence interval (* *p* < 0.05).

**Table 4:** Hazard ratios of RVO by sex between patients with SSNHL and comparison (subjects without SSNHL).

Sex	Male		Female	
	Comparison	SSNHL	Comparison	SSNHL
uHR (95% CI)	1.00 (ref)	1.18 (1.03–1.35) *	1.00 (ref)	1.03 (0.84–1.25)
aHR (95% CI)	1.00 (ref)	1.18 (1.03–1.34) *	1.00 (ref)	1.03 (0.84–1.25)

SSNHL—sudden sensorineural hearing loss; RVO—retinal vein occlusion; uHR—unadjusted hazard ratio; aHR—adjusted hazard ratio; CI—confidence interval (* *p* < 0.05).

## Data Availability

The datasets generated and/or analyzed for the current study are not publicly available due to the policy of the Korea National Health Insurance Service (KNHIS), but are available from the corresponding author upon reasonable request. The corresponding author had full access to all the data in the study and takes responsibility for both the integrity of the data and the accuracy of data analysis.

## References

[1] Wilson W.R., Byl F.M., Laird N. (1980). The Efficacy of Steroids in the Treatment of Idiopathic Sudden Hearing Loss. A Double-Blind Clinical Study. Arch. Otolaryngol..

[2] Byl F.M. (1984). Sudden Hearing Loss: Eight Years’ Experience and Suggested Prognostic Table. Laryngoscope.

[3] Fetterman B.L., Saunders J.E., Luxford W.M. (1996). Prognosis and Treatment of Sudden Sensorineural Hearing Loss. Am. J. Otol..

[4] Kuhn M., Heman-Ackah S.E., Shaikh J.A., Roehm P.C. (2011). Sudden Sensorineural Hearing Loss: A Review of Diagnosis, Treatment, and Prognosis. Trends Amplif..

[5] Chau J.K., Lin J.R., Atashband S., Irvine R.A., Westerberg B.D. (2010). Systematic Review of the Evidence for the Etiology of Adult Sudden Sensorineural Hearing Loss. Laryngoscope.

[6] Lee H., Lopez I., Ishiyama A., Baloh R.W. (2000). Can Migraine Damage the Inner Ear?. Arch. Neurol..

[7] Urban G.E. (1973). Reversible Sensori-Neural Hearing Loss Associated With Sickle Cell Crisis. Laryngoscope.

[8] Ostchega Y., Dillon C.F., Hughes J.P., Carroll M., Yoon S. (2007). Trends in Hypertension Prevalence, Awareness, Treatment, and Control in Older U.S. Adults: Data from the National Health and Nutrition Examination Survey 1988 to 2004. J. Am. Geriatr. Soc..

[9] Wild S., Roglic G., Green A., Sicree R., King H. (2004). Global Prevalence of Diabetes: Estimates for the Year 2000 and Projections for 2030. Diabetes Care.

[10] Centers for Disease Control and Prevention (CDC) (2007). State-Specific Prevalence of Cigarette Smoking among Adults and Quitting among Persons Aged 18–35 Years—United States, 2006. MMWR Morb. Mortal. Wkly. Rep..

[11] Marcucci R., Alessandrello Liotta A., Cellai A.P., Rogolino A., Berloco P., Leprini E., Pagnini P., Abbate R., Prisco D. (2005). Cardiovascular and Thrombophilic Risk Factors for Idiopathic Sudden Sensorineural Hearing Loss. J. Thromb. Haemost..

[12] Lin H.C., Chao P.Z., Lee H.C. (2008). Sudden Sensorineural Hearing Loss Increases the Risk of Stroke: A 5-Year Follow-up Study. Stroke.

[13] Kuo C.L., Shiao A.S., Wang S.J., Chang W.P., Lin Y.Y. (2016). Risk of Sudden Sensorineural Hearing Loss in Stroke Patients: A 5-Year Nationwide Investigation of 44,460 Patients. Medicine.

[14] Kim J.Y., Hong J.Y., Kim D.K. (2018). Association of Sudden Sensorineural Hearing Loss with Risk of Cardiocerebrovascular disease: A study Using Data from the Korea National Health Insurance Service. JAMA Otolaryngol. Head Neck Surg..

[15] Karia N. (2010). Retinal Vein Occlusion: Pathophysiology and Treatment Options. Clin. Ophthalmol..

[16] Rehak J., Rehak M. (2008). Branch Retinal Vein Occlusion: Pathogenesis, Visual Prognosis, and Treatment Modalities. Curr. Eye Res..

[17] Klein R., Klein B.E., Moss S.E., Meuer S.M. (2000). The Epidemiology of Retinal Vein Occlusion: The Beaver Dam Eye Study. Trans. Am. Ophthalmol. Soc..

[18] McIntosh R.L., Rogers S.L., Lim L., Cheung N., Wang J.J., Mitchell P., Kowalski J.W., Nguyen H.P., Wong T.Y. (2010). Natural History of Central Retinal Vein Occlusion: An Evidence-Based Systematic Review. Ophthalmology.

[19] Rogers S.L., McIntosh R.L., Lim L., Mitchell P., Cheung N., Kowalski J.W., Nguyen H.P., Wang J.J., Wong T.Y. (2010). Natural History of Branch Retinal Vein Occlusion: An Evidence-Based Systematic Review. Ophthalmology.

[20] Rim T.H., Oh J., Kang S.M., Kim S.S. (2016). Association between Retinal Vein Occlusion and Risk of Heart Failure: A 12-Year Nationwide Cohort Study. Int. J. Cardiol..

[21] Rim T.H., Kim D.W., Han J.S., Chung E.J. (2015). Retinal Vein Occlusion and the Risk of Stroke Development: A 9-Year Nationwide Population-Based Study. Ophthalmology.

[22] Kim J.Y., Ko I., Kim M.S., Kim D.W., Cho B.J., Kim D.K. (2020). Relationship of Chronic Rhinosinusitis with Asthma, Myocardial Infarction, Stroke, Anxiety, and Depression. J. Allergy Clin. Immunol. Pract..

[23] Kim J.Y., Kim Y.S., Ko I., Kim D.K. (2020). Association Between Burning Mouth Syndrome and the Development of Depression, Anxiety, Dementia, and Parkinson Disease. JAMA Otolaryngol. Head Neck Surg..

[24] Kim J.Y., Ko I., Kim D.K. (2019). Association of Obstructive Sleep Apnea with the Risk of Affective Disorders. JAMA Otolaryngol. Head Neck Surg..

[25] Kim J.Y., Ko I., Kim M.S., Yu M.S., Cho B.J., Kim D.K. (2019). Association of Chronic Rhinosinusitis with Depression and Anxiety in a Nationwide Insurance Population. JAMA Otolaryngol. Head Neck Surg..

[26] Fusconi M., Chistolini A., de Virgilio A., Greco A., Massaro F., Turchetta R., Benincasa A.T., Tombolini M., de Vincentiis M. (2012). Sudden Sensorineural Hearing Loss: A Vascular Cause? Analysis of Prothrombotic Risk Factors in Head and Neck. Int. J. Audiol..

[27] Lan M.Y., Shiao J.Y., Hsu Y.B., Lin F.Y., Lin J.C. (2011). A Preliminary Study on the Role of Inherited Prothrombotic Risk Factors in Taiwanese Patients with Sudden Sensorineural Hearing Loss. Eur. Arch. Otorhinolaryngol..

[28] Fusconi M., Chistolini A., Angelosanto N., Pignoloni P., Tombolini M., De Virgilio A., Pagliarella M., de Vincentiis M. (2011). Role of Genetic and Acquired Prothrombotic Risk Factors in Genesis of Sudden Sensorineural Hearing Loss. Audiol. Neurootol..

[29] Wong T.Y., Larsen E.K., Klein R., Mitchell P., Couper D.J., Klein B.E., Hubbard L.D., Siscovick D.S., Sharrett A.R. (2005). Cardiovascular Risk Factors for Retinal Vein Occlusion and Arteriolar Emboli: The Atherosclerosis Risk in Communities & Cardiovascular Health Studies. Ophthalmology.

[30] Rogers S., McIntosh R.L., Cheung N., Lim L., Wang J.J., Mitchell P., Kowalski J.W., Nguyen H., Wong T.Y., International Eye Disease Consortium (2010). The Prevalence of Retinal Vein Occlusion: Pooled Data from Population Studies from the United States, Europe, Asia, and Australia. Ophthalmology.

[31] Shin Y.U., Cho H., Kim J.M., Bae K., Kang M.H., Shin J.P., Nam E., Kang S.W., Epidemiologic Survey Committee of the Korean Ophthalmological Society (2016). Prevalence and Associated Factors of Retinal Vein Occlusion in the Korean National Health and Nutritional Examination Survey, 2008–2012: A Cross-Sectional Observational Study. Medicine.

[32] Guclu H., Ozal S.A., Pelitli Gurlu V., Özgün G.S., Özgün E. (2017). Increased Fibrinogen to Albumin Ratio in Ischemic Retinal Vein Occlusions. Eur. J. Ophthalmol..

[33] Cayir S., Kayabasi S., Hizli O. (2021). Predictor parameters for poor prognosis in patients with sudden sensorineural hearing loss: Fibrinogen to albumin ratio vs C-reactive protein to albumin ratio. Braz. J. Otorhinolaryngol..

[34] Glacet-Bernard A., Roquet W., Coste A., Peynègre R., Coscas G., Soubrane G. (2001). Central retinal vein occlusion and sudden deafness: A possible common pathogenesis. Eur. J. Ophthalmol..

[35] Lin R.J., Krall R., Westerberg B.D., Chadha N.K., Chau J.K. (2012). Systematic Review and Meta-Analysis of the Risk Factors for Sudden Sensorineural Hearing Loss in Adults. Laryngoscope.

[36] Kim S.H., Kim S.J., Im H., Kim T.H., Song J.J., Chae S.W. (2017). A Trend in Sudden Sensorineural Hearing Loss: Data from a Population-Based Study. Audiol. Neurootol..

[37] Cheung N., Klein R., Wang J.J., Cotch M.F., Islam A.F., Klein B.E., Cushman M., Wong T.Y. (2008). Traditional and Novel Cardiovascular Risk Factors for Retinal Vein Occlusion: The Multiethnic Study of Atherosclerosis. Investig. Ophthalmol. Vis. Sci..

[38] Lee J., Lee J.S., Park S.H., Shin S.A., Kim K. (2017). Cohort Profile: The National Health Insurance Service-National Sample Cohort (NHIS-NSC), South Korea. Int. J. Epidemiol..

[39] Kim D.K., Lee B.C., Park K.J., Son G.M. (2021). Effect of Obstructive Sleep Apnea on Immunity in Cases of Chronic Rhinosinusitis with Nasal Polyps. Clin. Exp. Otorhinolaryngol..

